# Management of the Axilla in the Era of Breast Cancer Heterogeneity

**DOI:** 10.3389/fonc.2018.00084

**Published:** 2018-04-04

**Authors:** Maïlys de Meric de Bellefon, Claire Lemanski, Angélique Ducteil, Pascal Fenoglietto, David Azria, Celine Bourgier

**Affiliations:** ^1^Institut Régional du Cancer de Montpellier (ICM), Montpellier, France; ^2^Institut de Recherche en Cancérologie de Montpellier (IRCM), INSERM U1194, Montpellier, France; ^3^Université de Montpellier, Montpellier, France

**Keywords:** breast cancer, heterogeneity, radiotherapy, axilla, molecular subtypes

## Abstract

Systemic cancer therapies take into account breast cancer (BC) heterogeneity by targeting pathways specifically involved in some BC subtypes. On the other hand, BC intrinsic radiosensitivity is poorly understood and studied. Hence, radiotherapy personalization in BC is still “work in progress”. In this review, we will summarize the existing data on the management of axillary lymph nodes in BC, the impact of BC radiotherapy on axillary management, the indications for axillary radiotherapy, and biomarkers to predict patients’ outcome (tumor control and late toxicities) after axillary irradiation.

## Introduction

Breast cancer (BC) is the most common cancer type in women worldwide. Treatment decision-making is based on the tumor histopathological features, tumor size, tumor grade, expression of hormone receptors (HRs) and HER2, lymphovascular invasion, and nodal involvement, to take into account BC morphological heterogeneity. Indeed, it has been proposed that although most tumors derive from a single mutated cell, tumor growth and aggressiveness are caused by sequential selection of more aggressive sublines that have acquired additional genetic alterations (genetic instability) (1). In 2000, BC diversity was further confirmed by the discovery that BC can be classified into different groups, according to their molecular phenotype, with different outcomes (2). Moreover, a better understanding of intratumor heterogeneity of primary BC and metastases provides clues about tumor resistance and/or genomic instability driver events, thus allowing the development of new and personalized treatments. Systemic treatment strategies are now proposed based on BC heterogeneity. Conversely, BC intrinsic radiosensitivity is poorly understood and studied, limiting the scope of personalized decision-making for locoregional treatment.

## Methods

The study objective was to identify recent literature data on axillary management and on predictive biomarkers for patients’ outcome (tumor control and late toxicities) after locoregional treatment of BC. To this aim, the Medline (Pubmed) and Cochrane Library databases were searched using the following search terms: “locoregional treatment”; “breast cancer”; “axillary nodes”; “radiotherapy”; “prognostic factors”; “predictive biomarkers”; “tumor control,” and “late toxicities”.

## Results

### Axillary Lymph Node Dissection (ALND) and Sentinel Lymph Node Biopsy (SLNB) in Early BC

After the publication of the results of the ACOSOG Z0011 and European Organization for Research and Treatment of Cancer (EORTC) 10981-22023 AMAROS clinical trials, ALND is mostly omitted for the management of early BC and replaced by SLNB (3, 4). Some limitations could be noticed in the ACOSOG Z011 trial, particularly the absence of assurance quality, the variations regarding the irradiation protocol (15% of patients received additional supraclavicular irradiation, prohibited by the protocol), the limited radiotherapy records (available only for 1/3 of the patients who were treated with radiotherapy), and the differences between radiotherapy treatment planning and delivery (3D-conformal vs. 2D) (5). Despite these limitations, the American Society of Breast Surgeons and the National Comprehensive Cancer Network guidelines have stated that SLNB is sufficient, even in the case of nodal involvement, for patients who meet all the ACOSOG Z0011 criteria: T1–2 BC, 1 or 2 positive lymph nodes without extracapsular extension, breast-conserving surgery followed by whole breast irradiation, and patient acceptance and completion of adjuvant systemic therapy (hormonal and/or cytotoxic) (6). In this selected population, the risk of axillary relapse is about 1–3% without any impact on disease-free survival (DFS) and overall survival (OS) (7–9). Similar recommendations were formulated based on the AMAROS trial results (10).

A recent report based on data from the Surveillance, Epidemiology, and End Results population study did not find any difference in breast cancer-specific survival in patients who underwent ALND or SLNB after adjustment for tumor stage, HRs status, and tumor grade (11). The study inclusion criteria were patients with T1-2 invasive BC and 1–2 positive lymph nodes (N1 only, patients with N0i+ and N1mi were not included). However, subgroup analyzes according to age (< or >50 years) and HRs status (positive or negative) showed that in the subgroup of women younger than 50 years and with a HRs-negative tumor, the BC-specific survival rate was higher in patients who underwent ALND than in those who had SLNB [hazard ratio (HR) = 0.70, HR = 0.026, 95% confidence interval (CI) = 0.51–0.96].

### Axillary Node Involvement: A Strong Prognostic Factor for BC OS

Axillary lymph node involvement is a major prognostic factor of BC outcome (12): the 5-year OS is 82.8% in node-negative BC, 73% in BC with 1–3 positive nodes, 45.7% in BC with 4–12 positive nodes, and 28.4% in BC with ≥13 positive nodes (13). The axillary lymph node status is a strong prognostic factor even in the case of microscopic nodal involvement with a 1.5-fold increase in the 5-year recurrence rate in patients with pN1mi BC compared with patients with node-negative BC (*p* = 0.02) (14).

### Optimal Number of Lymph Nodes to be Removed for Accurate Staging and Survival Prediction

When ALND is required, at least 10 lymph nodes should be removed (9). During SLNB, several sentinel and non-sentinel lymph nodes should be evaluated (1–8). As the occurrence of post-biopsy complications has been related to the number of removed lymph nodes, Ban et al. wanted to determine the optimal number of nodes to be collected for accurate prediction of the axillary lymph node status with minimal morbidity (15). By reviewing data on 328 patients with T1-2 BC who underwent SLNB, they found that all positive sentinel lymph nodes were identified in one of the first three lymph nodes removed from patients with node-positive BC. Therefore, they recommended that no more than four sentinel lymph nodes should be removed during SLNB. When only one sentinel lymph node was removed, recurrence-free survival was significantly worse (HR = 2.711; 95% CI = 1.110–6.622; *p* = 0.029) (16).

### The Role of Regional Lymph Node Irradiation (RNI)

In addition to ALND, RNI also has contributed to improve DFS and OS in patients with node-positive BC (17, 18). While RNI in ≥4 node-positive BC is the standard of care, its value in patients with 1–3 node-positive BC is still debated. The results of the MA.20 and EORTC 22922/10925 randomized trials showed a significant reduction in locoregional and distant relapses in patients who underwent RNI after a median follow-up of ≥9 years (19, 20). The reduction in BC recurrence rate was independent from the tumor molecular subtype. The contribution of RNI by site (supraclavicular, internal mammary chain, and/or axilla) on the improved outcome could not be distinguished because RNI encompasses at least two lymph node sites.

Finally, for early BC, the ESTRO consensus guidelines propose an atlas for the delineation of the node clinical target volume that includes the axillary levels (level 1, its visualization is influenced by scarring after ALND or SNB; level 2, dorsal to the minor pectoral muscle; level 3, or infraclavicular region; and level 4, or supraclavicular area), the interpectoral (or Rotter) nodes, and the internal mammary nodes (21). The aim of this atlas is to provide useful and reproducible guidelines for radiation oncologists [for a full description, see Ref. (21)].

### Impact of Tangent Radiation Fields and Subsequent Axillary Coverage on the Locoregional Outcome

The lowest part of the level I axillary region is usually covered by the breast tangent radiation fields, while the highest part of the level II and III axillary volumes are included in the supraclavicular irradiation field. If axillary irradiation is required, many authors consider that the usual breast tangent fields are not sufficient for optimal coverage of the level I and II axillary volumes, and an additional dedicated axillary field is needed (22).

The meta-analysis by Van Wely et al. showed that in patients with node-negative BC, whole breast irradiation significantly reduced axillary recurrence rate (relative risk = 0.32; *p* < 0.001) compared with patients without radiotherapy (23). As ALND is now rarely performed in the daily practice, the addition of direct axillary irradiation could be suggested to reduce regional recurrence. In the ACOSOG Z0011 trial (patients with cT1-2 N0-1 BC randomly divided in the ALND and SNB arms), radiotherapy with the high tangent field method that covers the lower axilla nodes was performed in 50 and 52.6% of patients in the ALDN and SBNB arms, supraclavicular irradiation in 21.2 and 16.9% of patients, respectively, and direct axilla irradiation was not allowed (5). After a median follow-up of 9 years, no significant difference in locoregional relapse was found between arms (24).

Following the AMAROS (4) and ACOZOG Z0011 trial results, additional ALND is now performed in fewer patients with sentinel node-positive BC. However, surgery de-escalation should be highly selective because some patients (more than 30%) could have ≥4 involved axillary nodes. Therefore, Haffty et al. suggested to perform breast irradiation with high tangent fields associated with full RNI in patients at high risk of aggressive BC (i.e., HRs-negative tumor, presence of lymphovascular invasion, multifocal disease, large tumor size, and at least three positive sentinel nodes) (25).

### Radiotherapy of the Axilla: for Which BC?

Axillary management differs according to the BC stage. In patients with early and pN1 BC, axillary irradiation is not recommended. The EBCTCG meta-analysis and the MA.20 and EORTC 22922/10925 trials showed a significant DFS improvement in patients with pN1 BC who received adjuvant RNI (19, 20). However, in these trials only irradiation of the level 3–4 axillary volumes and internal mammary nodes was considered.

In patients with locally advanced BC who undergo radical mastectomy, a meta-analysis of trials on radiotherapy of the chest wall and regional lymph nodes found that RNI and post-mastectomy irradiation improve DFS and OS (17, 18). Axillary nodes were covered by RNI in these trials. The risk of axillary recurrence significantly increases to 21–33% in patients with histologically positive axillary nodes with limited axillary dissection and without irradiation (7, 26, 27). In this setting, axillary node irradiation in addition to RNI is often indicated (expert agreement) (28–30).

In patients receiving neoadjuvant chemotherapy, some questions about axillary management, SLNB or ALND use, optimal SLNB timing (before or after neoadjuvant chemotherapy), and optimal irradiation volumes still need to be clearly addressed. Residual cancer cells in axillary nodes after neoadjuvant chemotherapy are a strong risk factor of locoregional relapse (31). A recent review by Pilewskie and Morrow suggested that ALND could be omitted in patients with cN0 HRs-positive and HER2-negative BC who undergo breast-conserving surgery. However, axillary management is still unclear for patients with triple negative or HER2-overexpressing BC (32). Regarding the node target volumes, Lemanski et al. and Rivera et al. suggested that node irradiation should be proposed to patients with ypN + BC, and axillary irradiation recommended for pN2-3 BC, or in the case of non-optimal number of harvested lymph nodes, or massive involvement of peri-nodal adipose tissue (33, 34).

### Prognostic Tools and Biomarkers of Patients’ Outcome

The IHC4 algorithm, which includes the protein expression level of estrogen and progesterone receptors, HER2, and Ki67, has been validated for the prediction of distant recurrences in patients with BC (35). Commercial mRNA-based gene signatures are also available for the prediction of distant recurrence [Oncotype Dx^®^/Genomic Health; MammaPrint^®^/Agendia; MapQuant Dx™ (GGI)/Ipsogen/QIAGEN; and ProSigna^®^/NanoString; EndoPredict^®^/Sividon/Myriad Genetics].

For locoregional management, nomograms have been developed to accurately estimate the probability of non-sentinel lymph node involvement in patients with positive SLNB. They use prognostic factors, such as tumor size, histology, lymphovascular invasion, total number of positive lymph nodes, metastasis size, and extracapsular extension (36–38). Lymphovascular invasion is an independent prognostic factor of BC-specific survival and distant metastasis-free survival in patients with node-negative BC (30, 39). Lymphovascular invasion is also a significant and independent prognostic factor in patients with pN1 BC and tumor size <2 cm. More recently, a meta-analysis showed that extranodal extension of a sentinel lymph node metastasis is significantly associated with worse patients’ outcome (40). The risk of recurrence and mortality is increased by twofold in the presence of extranodal extension.

Besides histological prognostic factors, some authors assessed whether specific BC molecular subtypes are associated with lymph node status. Unfortunately, no significant association was found between molecular subtypes and risk of positive nodes (41–43). Based on the hypothesis that metastatic cancer cells in axillary lymph nodes represent the most aggressive fraction of primary tumor cells, Feng et al. compared the gene expression profiles obtained by microarray analysis of matched axillary lymph node metastases and primary breast tumor with the aim of identifying predictive factors of patients’ outcome (44). They found 79 genes that were differentially expressed between matched samples and that could distinguish patients at low and high risk of distant recurrences. These results need to be confirmed in a prospective study with a large cohort. More recently, Paula et al. tried to identify prognostic markers in patients with pN0 and pN + BC (45) and found that the *PIK3R5* gene was differentially expressed in these two groups. However, they did not assess the correlation with the patients’ outcome. To date, no biomarker has been identified to stratify patients who will require ALND and axillary lymph node irradiation.

Regarding the locoregional outcome, Mamounas et al. recently reported that the Oncotype DX^®^ recurrence score is a significant predictor of locoregional recurrence in patients with node-positive (especially, more than four nodes) and estrogen receptor-positive BC treated with chemo- and endocrine therapy (46). They suggested that the Oncotype DX^®^ recurrence score could be combined with common clinicopathologic characteristics for more tailored radiotherapy.

### Prognostic Tools and Biomarkers of Radiotherapy Toxicity

De-escalation from ALND to SLNB significantly decreased the risk of arm lymphedema and improved the patients’ quality of life (47–49). Arm lymphedema and shoulder impairment can appear also after adjuvant breast radiotherapy, but less frequently than after ALND (50, 51). For instance, in the AMAROS trial, the 5-year lymphedema incidence was 11% after radiotherapy and 23% after surgery (*p* < 0.0001) (4). A study on the risk factors for lymphedema after cancer treatment in a large cohort of patients with BC showed that ALND and anthracycline-based chemotherapy significantly increased lymphedema occurrence (ALND: HR = 2.61; 95% CI = 1.77–3.84; anthracyclines: HR = 1.46; 95% CI = 1.04–2.04) (52). Few preclinical studies tried to identify predictive biomarkers of lymphedema risk. Newman et al. genotyped Tag single nucleotide polymorphisms for all genetic variations of genes involved in familial lymphedema and/or lymphangiogenesis in patients with BC who developed (*n* = 22) or not (controls, *n* = 98) arm lymphedema after surgery (53). They found that multiple SNPs within the *VEGFR2, VEGFR3*, and *RORC* genes were associated with lymphedema (*p* < 0.05). These preliminary results need to be confirmed in a larger cohort.

To date, no predictive biomarker of lymphedema occurrence has been validated in a large cohort.

## Conclusion

The characterization of BC intrinsic radiosensitivity is still in progress. In some BC population, such as patients with estrogen receptor-positive, pN + BC, the integration of the Oncotype DX^®^ recurrence score with common clinicopathologic characteristics could improve the prediction of the risk of locoregional recurrences and consequently allow a more tailored and comprehensive axillary management. However, axillary irradiation only concerns a small proportion of patients with BC. To date, no predictive tool or biomarker has been validated for the identification of patients who would require axillary irradiation. According to expert agreement, axillary node irradiation could be added to RNI for patients with high-risk BC after radical mastectomy and chest wall irradiation.

## Author Contributions

All the authors were involved in the conception/design of the work and provide approval for publication of the content more specifically. MB and CB contributed to drafting the work.

## Conflict of Interest Statement

The authors declare that the research was conducted in the absence of any commercial or financial relationships that could be construed as a potential conflict of interest.
